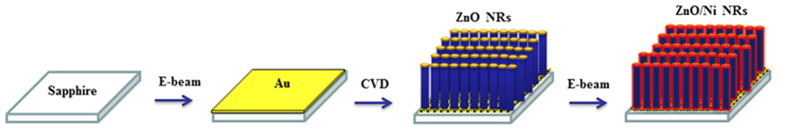# Zinc Oxide Nanorods Shielded with an Ultrathin Nickel Layer: Tailoring of Physical Properties

**DOI:** 10.1038/srep28561

**Published:** 2016-06-23

**Authors:** Devika Mudusu, Koteeswara Reddy Nandanapalli, Sreekantha Reddy Dugasani, Sung Ha Park, Charles W. Tu

**Affiliations:** 1 Department of Nanobio-Materials and Electronics, Gwangju Institute of Science and Technology, Gwangju 500712, Republic of Korea; 2 Department of Physics, School of Engineering and Technology, BML Munjal University, Sidhrawali, Gurgaon-122413, Haryana, India; 3 Department of Physics and Sungkyunkwan Advanced Institute of Nanotechnology (SAINT), Sungkyunkwan University, Suwon 440-746, Korea; 4 Department of Electrical and Computer Engineering, University of California, San Diego, La Jolla, CA 92093-0407, USA

## Abstract

We report on the development of Ni-shielded ZnO nanorod (NR) structures and the impact of the Ni layer on the ZnO NR properties. We developed nickel-capped zinc oxide nanorod (ZnO/Ni NR) structures by e-beam evaporation of Ni and the subsequent annealing of the ZnO/Ni core/shell nanostructures. The core/shell NRs annealed at 400 °C showed superior crystalline and emission properties. More interestingly, with the increase of annealing temperature, the crystallinity of the Ni shells over the ZnO NRs gradually changed from polycrystalline to single crystalline. The presence of the Ni layer as a polycrystalline shell completely hindered the light emission and transmission of the ZnO NR cores. Further, the band gap of ZnO NRs continuously decreased with the increase of annealing temperature.

Zinc oxide (ZnO) is a wide band gap (~3.37 eV) semiconductor material, showing an exciton binding energy of 60 meV. A highly transparent material for visible and near infrared light, ZnO exhibits good electrical conductivity, radiation hardness, and biocompatibility^1^. From this perspective, ZnO has gained much attention from researchers working in different fields, including electronics, optoelectronics, and bio-sensors^2^,^3^,^4^,^5^,^6^,^7^. Over the last two decades, researchers have developed the ZnO nanostructures using different synthetic techniques including: chemical vapor deposition (CVD), hydrothermal chemical solution, electrochemical, and pulsed laser deposition^8^,^9^,^10^. Further, researchers have subjected ZnO nanostructures to thorough testing for a variety of different applications (e.g., gas sensors^11^,^12^, solar cells^13^,^14^,^15^, light emitting diodes^16^,^17^, ultra-violet (UV) detectors^18^, and bio-sensors^19^). Further, the integration of ZnO nanostructures with other materials (e.g., either metals or semiconductors) as core/shell nanostructures has extended their potential applications to diverging fields.

In recent years, researchers have given great consideration to the realization of hetero-nanostructures by combining different materials as core/shell nanostructures due to their collective properties (as compared to individual structures). In particular, ZnO nanostructures with metallic shell-layers (Ag, Au, Ni, Co) have exhibited interesting device properties as: photocatalysts^20^,^21^,^22^, photoelectrochemical anodes^23^,^24^, sensors^25^, DNA detectors^26^, and dye sensitized electrodes^27^,^28^,^29^. On the other hand, ZnO nanostructures passivated with other semiconductors (TiO_2_, CdS, ZnS, InN, SiO_2_) have also shown multifunctional applications^30^,^31^,^32^,^33^,^34^. Significantly, in all cases, the passivation layer over ZnO nanostructures plays different roles, including: surface defect states neutralizer, environmental or chemical protector, carrier separator, absorbability enhancer, and electrical signal amplifier^35^. However, the growth or deposition of a passive-layer as a second-order structure strongly influences the ZnO nanostructures properties due to its own inherent properties (particularly structural and optical), along with the interfacial layer formed between the ZnO and the passive-material^36^. Thus, we consider it essential to understand and explore the impact of a passive-layer on the physical properties of the ZnO nanostructures. To do this, we have chosen nickel (Ni) as a passivation layer due to its cost-effectiveness and easy material processability (as compared to Au and Ag)^37^. Furthermore, Ni also has very good electrical conductivity, capable of good Ohmic contact behavior with ZnO. As such, ZnO/Ni core/shell NR structures could be adopted for ether dye-sensitized solar cells or photoelectrochemical devices as anodes^38^.

In the current paper, we report on the deposition of an ultrathin (10-nm thick) Ni layer over ZnO NRs, as well as its impact on the physical properties (particularly light emission and transmission) of aligned ZnO NRs. We developed vertically-aligned ZnO NRs by chemical vapor deposition; then, we deposited an ultrathin Ni layer by e-beam evaporation. In order to understand the influence of the crystalline characteristics of the Ni layer on the optical properties of the ZnO NRs, we also annealed the ZnO/Ni core/shell structures at different temperatures. From these studies, we observed that the ZnO/Ni core/shell NR structures annealed at 400 °C possessed significant crystalline and light emission properties, being prerequisites for the development of efficient photoelectrochemical devices.

## Results and Discussion

**Figure 1:**
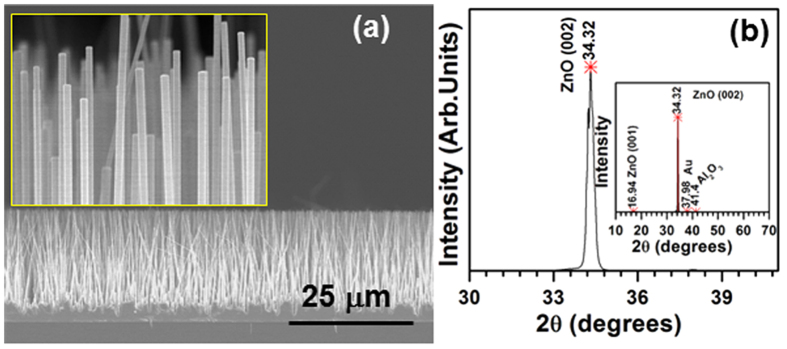
(**a**) Low magnification FESEM cross-section image (inset shows its high magnification image); and (**b**) XRD profile recorded between 30–40° (inset shows its complete profile) of as-grown ZnO NR structures.

Figure 1(a) shows field emission scanning electron microscopic (FESEM) cross-section images of as-grown ZnO NRs, revealing their vertically-aligned growth and uniform surface morphology. These NRs consisted of an average length of about 30 μm, while their diameters varied between 50 and 100 nm. Figure 1(b) shows x-ray diffraction (XRD) studies of all the as-grown ZnO NRs, preferentially-oriented along the <001> direction and having a full width at half maximum (FWHM) value of 0.25°; the calculated d-spacing value for the major peak diffracted at 2θ = 34.32° is 0.2611 nm, nearly matching with the hexagonal ZnO data (JCPDS data of ZnO #36-1451). We evaluated the dislocation density (DD) of the nanostructures using the following equation, where b_s_ was the Burgers vector sizes of the screw-type dislocations (~0.5185 nm) and θ was the angle between the reciprocal vector and (001) surface normal:


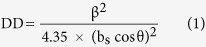


We found the DD for the as-grown ZnO NRs to be 1.6 × 10^9^ cm^−2^. We evaluated the lattice constant ‘c’ of the ZnO NRs using the d-spacing value of the (002) peak, found to be 0.522 nm. A minor peak diffracted at 16.94° (d = 0.523 nm) belonged to the ZnO (001) plane, whereas the other peaks diffracted at 37.98 and 41.4° belonged to the Au catalyst and sapphire substrate, respectively. These results indicated that though the RTCVD-grown ZnO NRs had uniform surface morphology and excellent crystalline quality, the structures contained a large number of dislocations.

**Figure 2:**
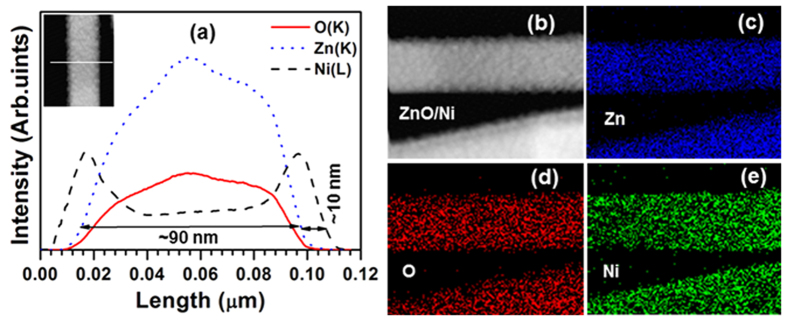
Elemental mapping of (**a**) line (inset shows STEM image); (**b**) STEM image of ZnO/Ni core/shell nanostructure where the actual scanning occurred; and (**c–e**) the corresponding area scanned images for the Zn, O, and Ni elements.

In order to find the variation in the chemical composition of the ZnO NRs, we analyzed the as-grown ZnO/Ni core/shell NRs by energy dispersive spectroscopy (EDS) with the help of STEM. Figure 2(a) shows the spatial distributions of the elemental composition across the ZnO/Ni core/shell NRs, obtained by line-scan elemental mappings of Zn, O, and Ni. The profiles of Zn (blue) and O (red) showed a broadband-like peak at the center, while the profile of Ni (black) showed sharp peaks at both sides of the Zn and O profiles with a broad valley at the center. From these analyses, we extracted the average diameter of the ZnO NR cores and the thickness of the Ni shells as ~90 and ~10 nm, respectively. We also confirmed these results by elemental mapping of the ZnO/Ni core/shell NRs, as shown in Fig. 2(b), clearly emphasizing that the as-grown ZnO/Ni core/shell NRs purely consisted of Zn, O, and Ni elements with appropriate ratios, while also possessing a well-defined composition profile with an excellent interface between the ZnO and Ni components.

We examined the changes in the crystallinity of the as-grown and annealed ZnO/Ni core/shell NRs by XRD, providing a detailed descriptive report about these issues elsewhere^39^. In brief, the as-grown core/shell nanostructures exhibited similar structural characteristics as bare-ZnO NRs. However, these structures showed an additional diffraction peak, belonging to the (002) orientation of hexagonal Ni (JCPDS data of ZnO #36-1451, Ni #45-1027, Ni2O3 #14-0481). Upon annealing, noticeably two more diffraction peaks emerged (see Supporting Information, Figure SI-1) belonging to hexagonal ZnO (110) and hexagonal Ni_2_O_3_ (202) orientations. Though the observed intensities of these additional peaks gradually increased with increasing annealing temperature, the existence of these phases was very low since the relative intensity ratio between ZnO (110) [Ni_2_O_3_ (202)] and ZnO (002) of ZnO/Ni core/shell NRs annealed at 400 °C was 0.03% [0.024%].

**Figure 3:**
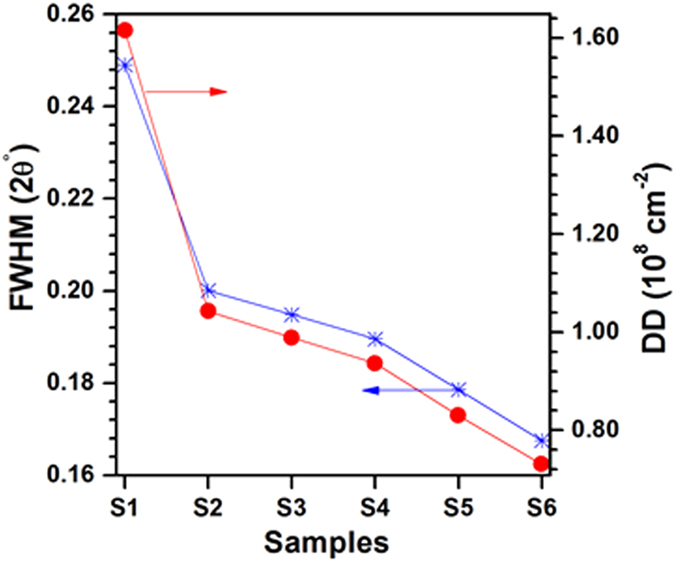
Variation of the FWHM and dislocation density (DD) of ZnO and ZnO/Ni core/shell structures (S1-ZnO; S2-as-grown ZnO/Ni; S3-100 °C; S4-200 °C; S5-300 °C; and S6-400 °C annealed core/shell structures).

Figure 3 shows the variation of the FWHM and DD values of the (002) ZnO peak as a function of annealing temperature, indicating that after the deposition of the Ni layer over the ZnO NRs, both the FWHM and DD values slightly decreased from 0.25 to 0.20° and 1.6 × 10^9^ to 1.0 × 10^9^ cm^−2^, respectively^40^,^41^. While increasing the annealing temperature of the ZnO/Ni core/shell NRs, both these parameters gradually decreased, with the structures annealed at 400 °C showing the lower values of 0.17° and 7.3 × 10^8^ cm^−2^. These observations emphasized that the deposition of the Ni layer over the ZnO NRs and their annealing significantly passivated the surface defects present on the ZnO core structures; as a result, the crystalline quality of the ZnO NRs (i.e., ordering of crystallites) strongly enhanced^42^.

**Figure 4:**
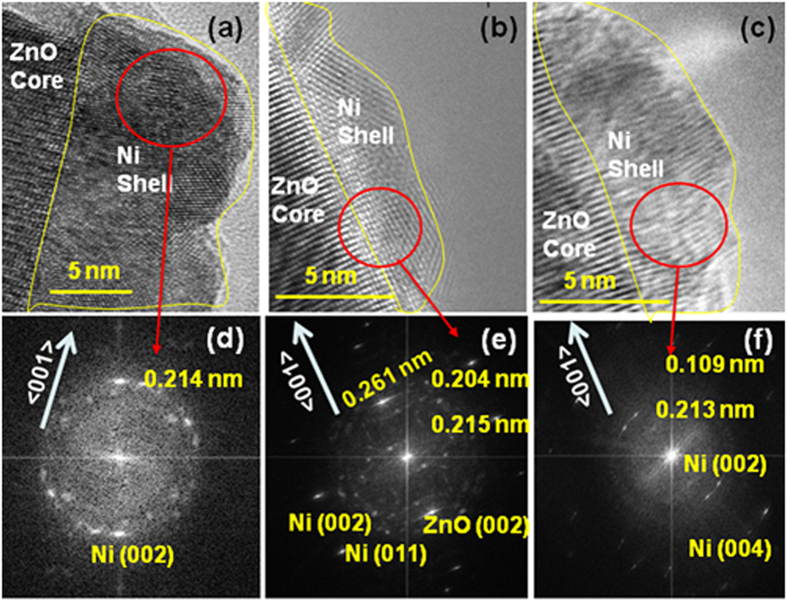
HRTEM images of (**a**) as-grown; (**b**) 200 °C; (**c**) 400 °C annealed ZnO/Ni core/shell nanostructures; and (**d–f**) their corresponding FFT images recorded at the interfaces of the ZnO and Ni structures.

In order to understand the interfacing characteristics of the Ni layer with the ZnO NRs, we examined the untreated and treated ZnO/Ni core/shell NRs with high resolution transmission electron microscopy (HRTEM), shown in Fig. 4; these images show that the as-deposited Ni nano-layer over the ZnO NRs had uniform surface morphology, while we found its thickness to be ~10 nm. The fast Fourier transformation (FFT) analysis of the HRTEM image, Fig. 4(d), confirmed that the as-deposited Ni nano-layer had polycrystalline nature. However, the Ni layer consisted of preferentially-oriented crystallites, oriented along the <001> direction. Thus, the as-deposited Ni structures on the hexagonal facets of the ZnO NRs were composed of fine hexagonal polycrystalline nanocrystals with a preferential growth direction of <001>. Figures 4(b,c,e,f) show the HRTEM and their corresponding FFT images of the annealed ZnO/Ni nanostructures at 200 and 400 °C, respectively. The ZnO/Ni nanostructures annealed at 200 °C still consisted of a polycrystalline Ni layer over single crystalline ZnO NRs^39^. However, with the further increase of annealing temperature to 400 °C, the polycrystalline Ni-layer converted to single-crystalline structures, as shown in Fig. 4(f). On the other hand, we observed the presence of Ni_2_O_3_ phase, clearly noticed by electron diffraction studies (see Figure SI-2), exclusively over ZnO/Ni core/shell NRs surfaces.

**Figure 5:**
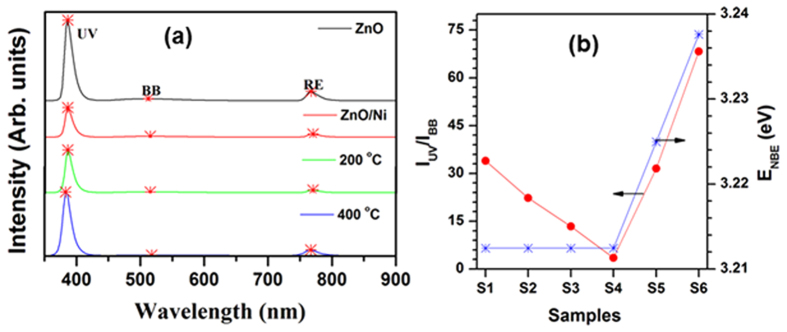
(**a**) Cathodoluminescence spectra; and (**b**) variation of I_UV_/I_BB_ intensity ratio and UV peak position (E_NBE_) of the ZnO and ZnO/Ni structures (S1-ZnO; S2-as-grown ZnO/Ni; S3-100 °C; S4-200 °C; S5-300 °C; and S6-400 °C annealed core/shell structures).

Figure 5(a) shows the cathodoluminescence (CL) spectra of pure ZnO NRs, as well as untreated and treated ZnO/Ni core/shell nanostructures. The pure ZnO NRs exhibited a strong emission peak at 386 nm and two weak peaks at 512 and 767 nm. We attributed these peaks to the transition of excited electrons from the conduction band: i) to the valence band (near band edge or ultra-violet emission, NBE or UV); ii) through various interstitial states (broadband, BB)^43^; or iii) to the second harmonic emission of NBE (RE, red emission), respectively^44^. After deposition of the Ni layer and increasing the annealing temperature up to 200 °C, the NBE peak position remained the same, while the I_UV_/I_BB_ peak ratio decreased. After further increasing the annealing temperature, both the NBE peak position and the I_UV_/I_BB_ ratio significantly increased, as shown in Fig. 5(b), emphasizing that deposition of an ultrathin Ni layer over the ZnO NRs drastically hindered their light emission characteristics (continuing up to the annealing temperature of 200 °C). We attributed the significant improvement in the emission properties and widening NBE band of the ZnO NRs annealed at higher temperatures (>200 °C) to the formation of single-crystalline Ni crystals and a decrease of surface density states^45^. As a result, ZnO/Ni core/shell nanostructures annealed at 400 °C possessed excellent CL emission properties^42^.

**Figure 6:**
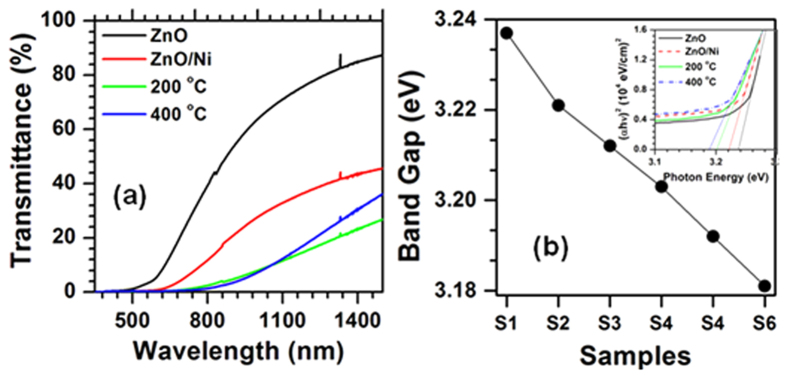
(**a**) UV-Vis-NIR spectra of the ZnO, ZnO/Ni, 200 °C, 400 °C treated structures; and (**b**) variation of E_g_ of as-grown and ZnO/Ni core/shell NRs (S1-ZnO; S2-as-grown ZnO/Ni; S3-100 °C; S4-200 °C; S5-300 °C; and S6-400 °C annealed core/shell structures) (inset shows the (αhν)^2^ vs E plots of the ZnO, ZnO/Ni, 200 °C, 400 °C treated structures).

Figure 6(a) shows optical transmittance plots of as-grown and annealed ZnO/Ni core/shell NRs. After the deposition of the Ni metallic layer, the transmittance of the ZnO structures drastically decreased, while further decreasing with annealing temperature up to 200 °C. Above this temperature, the transmittance of the structures gradually improved. Variation in the transmittance of the structures mainly depended on the morphology of the ZnO/Ni structures, since the as-deposited and annealed structures up to 200 °C had a uniform metallic shield that reduced the light penetration as well as emission, resulting in a strong impact on their light transmission.

As observed in the XRD studies, the formation of single crystalline Ni phase (above 200 °C) played a crucial role in the improvement of light transmission, since the formation of crystalline phase with smaller openings gradually allowed (Figure SI-3) the light through the shells. At near the fundamental absorption edge, the absorption coefficient (α = ln(100/T)/t) satisfied the following equation, where T was the transmittance; t was the thickness (height of the NRs); A was the proportional constant; E was the photon energy; E_p_ was the phonon energy; and E_g_ was the optical band gap:


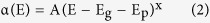


For direct transition (E_p_ = 0), x was equal to 1/2 for allowed transition and 3/2 for forbidden transition^46^,^47^. For indirect transition, x = 2 for allowed transition and 3 for forbidden transition. In the present case, the square of the absorption coefficient on the incident photon energy, at above the fundamental absorption edge (α_c_), was a straight line, since above α_c_ it satisfied α^2^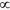 E (=hν) relation (inset of Fig. 6(b)). Thus, the transition between valence band and conduction band near the fundamental band edge was direct. Therefore, we evaluated the energy band gap (E_g_) of the structures using the equation (αhν)^2^ ~ hν−E_g,_, where ‘h’ was Planck’s constant and ‘ν‘ was the incident photon frequency; the variation of the optical band gap of the structures with annealing temperature is shown in Fig. 6(b).

The as-grown ZnO NRs showed an optical band gap of ~3.24 eV, slightly lower (60 meV) than the bulk ZnO^1^. After the deposition of the Ni metallic layer, it decreased to 3.22 eV, while further decreasing with the increase of annealing temperature. We strongly attributed these changes in the optical band gap of the ZnO nanostructures to the dislocation density of the core nanostructures. As observed in the XRD studies, gradual improvement in the crystalline quality of the ZnO NRs due to the eradication of dislocations by Ni atoms probably led to lower values in the optical band gap^48^. On the other hand, according to the orbital hybridization theory, the formation of antibonding 3d-triplet states (with higher energies lying close to the conduction band of the ZnO) led to lower values in the band gap^49^. Therefore, surface passivation of the ZnO NRs by Ni atoms probably led to lower values in the overall band gap.

In summary, we investigated the impact of Ni-capping on the physical properties of vertically-aligned ZnO NR structures. We synthesized vertically-aligned ZnO NRs using the vapor-liquid-solid method with the help of a gold catalyst, while we deposited the Ni nano-layer using an e-beam evaporator. Upon annealing, the quality of the ZnO/Ni structures clearly improved due to the eradication of structural defects and/or passivation of surface defect states of the ZnO NRs. However, the light emission and transmission of the ZnO NRs drastically decreased with the deposition of the Ni layer and subsequent annealing up to the temperature of 200 °C. The structures annealed at higher temperatures exhibited significant improvement in the emission as well transmission of light. The band gap of ZnO NRs gradually decreased with the increase of annealing temperature of the ZnO/Ni core/shell nanostructures. Based on these results, we emphasize that Ni-capped ZnO nanostructures annealed at higher temperatures could be utilized for different device applications, particularly for photoelectrochemical water-splitting devices due to their better structural and light emission properties.

## Experimental Procedure

### Synthesis

We achieved ZnO/Ni core/shell nanorod structures in three-steps, schematically-represented in Fig. 7. Initially, we cleaned c-plane Al_2_O_3_ substrates (both sides polished sapphire substrates) by using ethanol, acetone, and deionized water under ultrasonic agitation for 10 min; then, we deposited the Au catalyst with a thickness of ~3 nm using e-beam evaporation. We synthesized the ZnO NRs using rapid thermal chemical vapor deposition (RTCVD) on Au-coated substrates by the vapor-liquid-solid (VLS) method. We carried out the growth of the ZnO NRs for 10 min at a temperature and pressure of 950 °C and 20 Torr, respectively. Here, we used Ar as a carrier gas and O_2_ as a partial source of oxygen with flow rates of 100 and 2 sccm, respectively. Then, we carried out the deposition of the Ni layer on the ZnO NRs using an e-beam evaporator at room temperature. Finally, we annealed the as-grown ZnO/Ni core-shell NRs at different temperatures, varied from 100 to 400 °C in the RTCVD system under a vacuum of 10^−3^ Torr for a fixed time of 5 min.

### Characterization

We studied the structural properties of as-grown and annealed ZnO/Ni structures by powder X-ray diffraction (XRD) using Cu Kα_1_ radiation in the range of 10–70°. We used field emission scanning electron microscopy (FESEM) and transmission electron microscopy (TEM) for the examination of surface morphology. We used scanning TEM (STEM) and energy dispersive spectroscopy (EDS) embedded with TEM for chemical composition analyses. We analyzed the crystalline characteristics of the Ni layers with high resolution transmission electron microscopy (HRTEM). We studied optical properties (e.g., emission and transmission) of the ZnO/Ni core/shell nanostructures by measuring their light emission and transmission versus wavelengths using mono-cathodoluminescence (CL) attached with FESEM and UV-Vis-NIR spectrophotometer. We estimated the light transmission of the structures in the wavelength range of 300–1500 nm with an incident light perpendicular to the growth direction of the ZnO NRs.

## Additional Information

**How to cite this article**: Mudusu, D. *et al*. Zinc Oxide Nanorods Shielded with an Ultrathin Nickel Layer: Tailoring of Physical Properties. *Sci. Rep.* **6**, 28561; doi: 10.1038/srep28561 (2016).

## Supplementary Material

Supplementary Information

## Figures and Tables

**Figure 7:**