# Supplementary material for: Zinc Oxide Nanorods Shielded with an Ultrathin Nickel Layer: Tailoring of Physical Properties
Source: Sci Rep. 2016 Jun 23;6:28561. doi: 10.1038/srep28561 (PMC4917843; doi:10.1038/srep28561)
Supplement: Supplementary Information [file srep28561-s1.pdf]

Supporting Information

Zinc Oxide Nanorods Shielded with an Ultrathin Nickel Layer: Tailoring of Physical Properties

Devika Mudusu,[1] Koteeswara Reddy Nandanapalli,[1,2] Sreekantha Reddy Dugasani,[3] Sung Ha Park,[3] and Charles W. Tu[1,4]

[1]Department of Nanobio-Materials and Electronics, Gwangju Institute of Science and Technology, Gwangju 500712, Republic of Korea

[2]Department of Physics, School of Engineering and Technology, BML Munjal University, Sidhrawali, Gurgaon-122413, Haryana, India

[3]Department of Physics and Sungkyunkwan Advanced Institute of Nanotechnology (SAINT), Sungkyunkwan University, Suwon 440-746, Korea

[4]Department of Electrical and Computer Engineering, University of California, San Diego, La Jolla, CA 92093-0407, USA

Figure SI-1: XRD spectrum of as-grown and annealed ZnO/Ni structures:

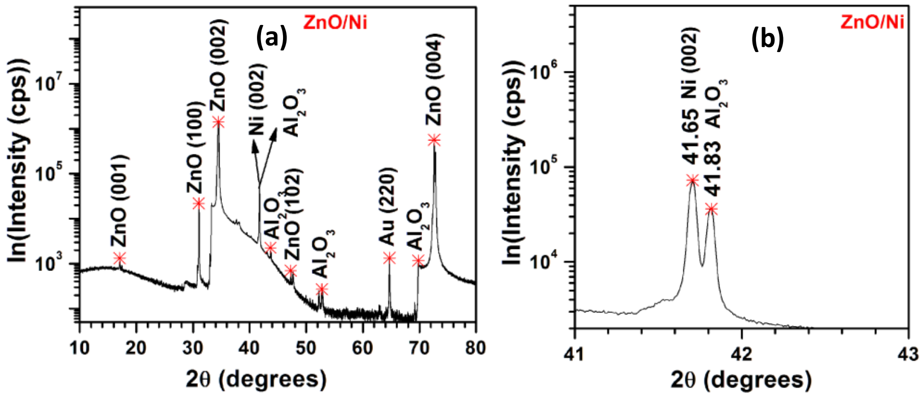

(a) XRD spectrum of as-grown ZnO/Ni structure; (b) enlarged spectrum;

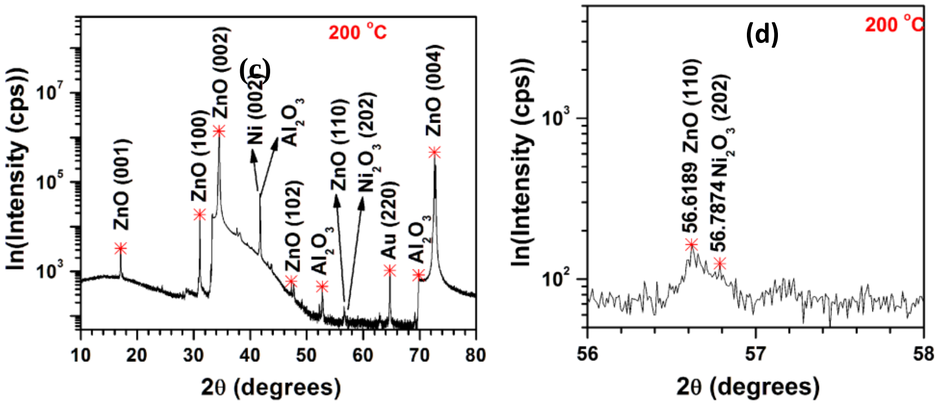

(c) XRD spectrum of annealed ZnO/Ni structure at 200°C; (d) enlarged spectrum;

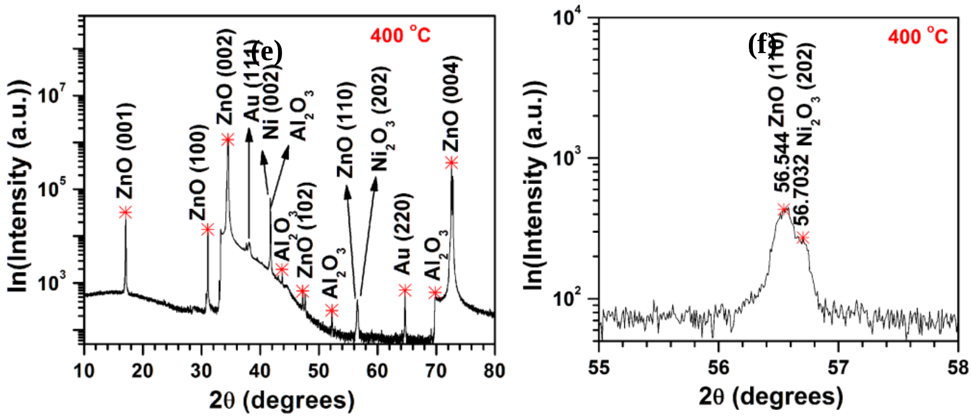

(e) XRD spectrum of annealed ZnO/Ni structure at 400°C; (f) enlarged spectrum.

Figure SI-2: SAED images of as-grown and annealed ZnO/Ni core/shell nanostructures.

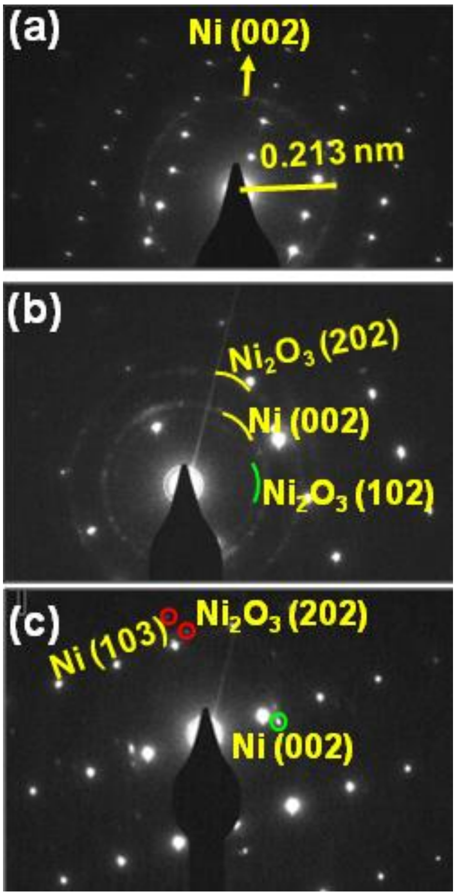

Formation of single crystalline Ni phase and Ni_2O_3 phase

Figure SI-3: HRTEM images of annealed ZnO/Ni NRs at 400 ^oC.

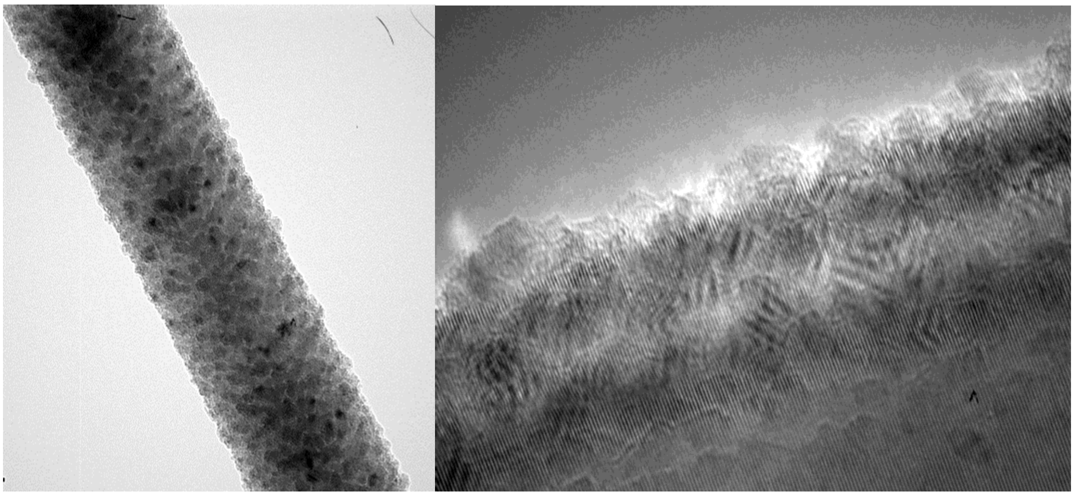

Appearance of surface of ZnO/Ni NRs annealed at 400 ^oC.
